# The significance of programmed cell death ligand 1 expression in resected lung adenocarcinoma

**DOI:** 10.18632/oncotarget.14851

**Published:** 2017-01-27

**Authors:** Shafei Wu, Xiaohua Shi, Jian Sun, Yuanyuan Liu, Yufeng Luo, Zhiyong Liang, Jinghui Wang, Xuan Zeng

**Affiliations:** ^1^ Department of Pathology, Peking Union Medical College Hospital, Beijing, China; ^2^ Department of Medical Oncology, Beijing Tuberculosis and Thoracic Tumor Research Institute, Beijing Chest Hospital, Capital Medical University, Beijing, China

**Keywords:** lung adenocarcinoma, programmed cell death ligand 1, immunohistochemistry, in situ hybridization

## Abstract

**Background:**

Lung adenocarcinoma (AD) is a common variant of non-small cell lung cancer (NSCLC). Programmed cell death protein 1/programmed cell death ligand 1 (PD1/PD-L1) are promising immunotherapy targets and its expression may be an important biomarker of predicting clinical response. In this study, we evaluated PD-L1 expression in conjunction with clinicopathological characteristics and outcomes in resected lung adenocarcinoma.

**Results:**

This study included 133 cases of lung adenocarcinoma. PD-L1 expression rate in lung adenocarcinoma was 16.5% at the mRNA level and 13.5% at the protein level, and the kappa coefficient of the two examination methods was 0.824 (*P* = 0.219, highly correlated). PD-L1 was highly expressed in male patients and smokers with lung adenocarcinoma (*P* = 0.019 and 0.002, respectively), while no associations were identified between PD-L1 expression and age, tumor size, clinical stage, positive pleural invasion, lymph node metastasis, or therapy methods. Overexpression of PD-L1 was a significant indicator of shorter recurrence free survival time and overall survival (*P* = 0.000 and 0.000, respectively). Multivariate analysis revealed that PD-L1 expression was an independent risk factor for poor recurrence free survival and overall survival (*P* = 0.009 and 0.016, respectively).

**Materials and Methods:**

Expression of PD-L1 was examined with immunohistochemistry, using the VENTANA PD-L1 (SP263) rabbit monoclonal antibody. mRNA levels of PD-L1 were evaluated using *in situ* hybridization.

**Conclusions:**

PD-L1 overexpression is more frequently observed in male patients and smokers in lung adenocarcinoma. PD-L1 expression is an indicator of worse prognosis in surgically resected lung adenocarcinoma patients.

## INTRODUCTION

Lung adenocarcinoma is a common variant of non-small cell lung cancer (NSCLC). In recent years, there have been dramatic advances in the treatment of lung adenocarcinoma because of the development of therapies targeting driver oncogene alterations, for example, drugs targeting the epidermal growth factor receptor (EGFR) mutation or the anaplastic lymphoma kinase (ALK) fusion [1, 2]. However, most patients will inevitably develop acquired resistance to the targeted therapy [3–5]. Therefore, exploration into new lung cancer treatments is ongoing. Recently, immune check-point inhibition therapy has shown promising results in several kinds of malignant tumors, including NSCLC [6].

Immune checkpoints are inhibitory pathways that can maintain self-tolerance and protect peripheral tissues by modulating immune response [7]. Overexpression of immune checkpoint proteins in tumor cells or tumor-infiltrating lymphocytes (TILs) can help tumor cells escape surveillance by the immune system. Immune checkpoint inhibitor therapy can block the ligand- receptor interaction and activate a T cell immune response to attack the tumor cells. Programmed cell death protein 1/programmed cell death ligand 1 (PD-1/PD-L1) is a major immune checkpoint signaling pathway. The binding of PD-L1 to PD-1 can block the immune response of T cells to the tumor cells. There are several ongoing clinical trials targeting NSCLC with PD-L1 protein. Pre-released data has shown that in unselected patients treated with anti–PD-1/PD-L1 antibodies, roughly 20% had a meaningful response to therapy [8]. PD-L1 protein expression is reported to be a potential predictor of therapy response [9]. Careful selection of the specific population who will benefit from the targeted therapy is urgent. There are several commercial immunohistochemical (IHC) antibodies that have been designed as accompanying diagnostic tools for different clinical trials. However, owing to diverse examination methods and cutoff values, a unified standard examination method is needed for the selection of suitable targeted populations.

In this study, we evaluated PD-L1 expression using immunohistochemistry at the protein level and by *in situ* hybridization at the mRNA level. Furthermore, we compared the expression of PD-L1 with clinicopathological characteristics and outcomes in lung adenocarcinoma.

## RESULTS

### Clinicopathological characteristics of lung adenocarcinoma

The clinicopathological characteristics of the lung adenocarcinoma patients are summarized in Table 1. The median age was 58.94 years old (range, 32–84). Fifty-three (39.8%) patients were male and 80 were female. Ninety-seven (74.0%) had never smoked and 34 were smokers. The average tumor size was 3.2 cm (range, 1.5–7.0 cm). Tumors of stages I, II, III, and IV were observed in 65 (48.9%), 16 (12.0%), 42 (31.6%), and 10 (7.5%) cases, respectively. Post-operative therapy was performed in 65 patients: 64 patients received chemotherapy; 6 were exposed to radiation therapy, and 5 received both types of therapy.

**Table 1:** Relationship between PD-L1 IHC expression and clinicopathological characteristics of lung adenocarcinoma patients

Characterisitcs (*N* = 133)	No. of patients (Percentage)	PD-L1 IHC		
		Positive (*N* = 18)	Negative (*N* = 115)	*p*-value
**Age**				1.000
> = 70	39 (29.3%)	5	34	
< 70	94 (70.7%)	13	81	
**Gender**				0.019
Female	80 (60.2%)	6	74	
Male	53 (39.8%)	12	41	
Smoking*				0.002
Smokers	34 (26.0%)	10	24	
Non- smokers	97 (74%)	7	90	
**Tumor Size (cm)**				0.613
> 3	53 (39.8%)	6	47	
< = 3	80 (60.2%)	12	68	
**Stage**				0.067
Early (I–II)	81 (60.9%)	7	74	
Advanced (III–IV)	52 (39.1%)	11	41	
Invasion of pleural^*^				0.553
Yes	92 (70.2%)	14	78	
No	39 (29.8%)	4	35	
**Node metastasis**				0.439
Yes	53 (39.8%)	9	44	
No	80(60.2%)	9	71	
**Histological subtype**				0.000
Acinar+lepdic	81 (60.9%)	4	77	
Others	52 (39.1%)	14	38	

Abbreviations: AD, adenocarcinoma; PD-L1, programmed cell death ligand 1; IHC, immunohistochemistry.

*Two patients lost of follow-up; **Two patients lost of follow-up.

The growth pattern of lung adenocarcinoma was classified into lepidic (16 tumors), acinar (65), mucinous (8), solid (18), papillary (19), and micropapillary (7) patterns. Pleural involvement was identified in 92 cases. Positive node metastasis was present in 53 cases.

### Comparison of PD-L1 expression examined by immunohistochemistry and RNA *in situ* hybridization methods

**Figure 1:**
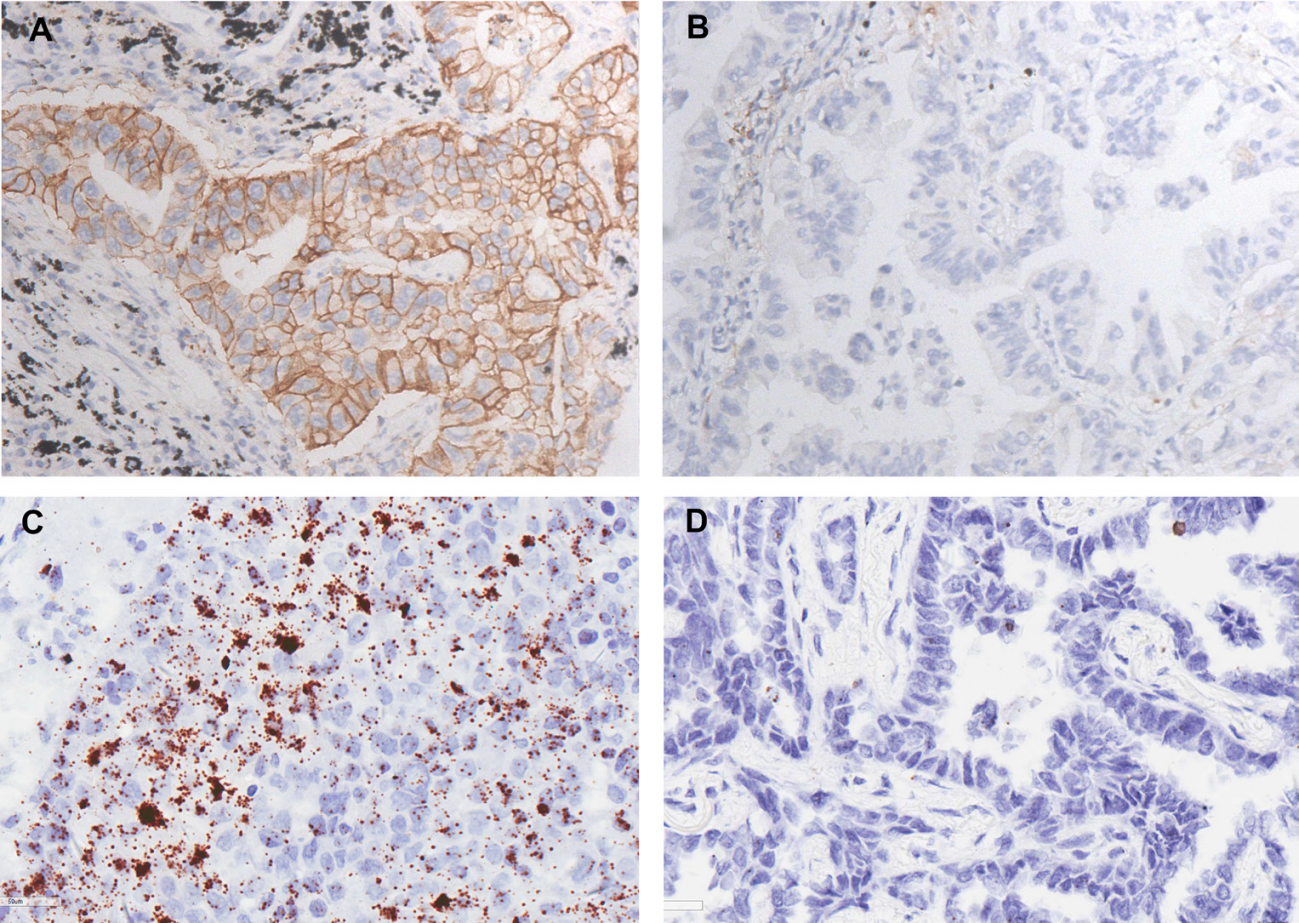
Representative results of PD-L1 expression in lung adenocarcinoma (**A**) Positive result of PD-L1 examined via immunohistochemistry method. (×40) (**B**) Negative result of PD-L1 examined via immunohistochemistry method. (×40). (**C**) Positive result of PD-L1 examined via RNA *in situ* hybridization method. (×40). (**D**) Negative result of PD-L1 examined via RNA *in situ* hybridization method. (×40)

Of the 133 cases of lung adenocarcinoma examined in this study, the PD-L1 expression rate in lung adenocarcinoma detected by IHC and ISH was 13.5% (18/133) and 16.5% (22/133), respectively. The two techniques were consistent in identifying 110 cases as PD-L1 negative, and 17 cases as PD-L1 positive. Representative cases of IHC and ISH results are shown in Figure 1. The concordance between IHC and mRNA ISH results was near perfect at 95.5% (127/133), with a κ-coefficient of 0.824 (Table 2). No significant difference between the two methods was detected with the McNemar-Bowker test (*P* = 0.219).

**Table 2:** Comparison of immunohistochemistry and in situ RNA detection methods for evaluation of PD-L1 expression

		PD-L1 IHC		Kappa	*p*-value
		−	+		
**PD-L1**	–	110	5	0.824	0.219
**RNA ISH**	+	1	17		

Abbreviations: PD-L1, programmed cell death ligand 1; IHC, immunohistochemistry; ISH, *in situ* hybridization.

### PD-L1 expression and its association with clinicopathological characteristics

Expression of PD-L1 was significantly higher in male patients than in female patients (*P* = 0.019); in smokers than non-smokers (*P* = 0.002); and in solid, papillary, or micropapillary growth pattern tumors compared to acinar and lepidic growth pattern tumors (*P* = 0.000). No significant association was detected between expression of PD-L1 and patient age (≥ 70 versus < 70 years, *P* = 1.000), tumor size (≤ 3 cm versus > 3 cm, *P* = 0.613), clinical stage (I + II versus III+IV, *P* = 0.067), pleural involvement (*P* = 0.553), or lymph node metastasis (*P* = 0.439).

### Prognostic significance of PD-L1 expression in lung adenocarcinoma

**Figure 2:**
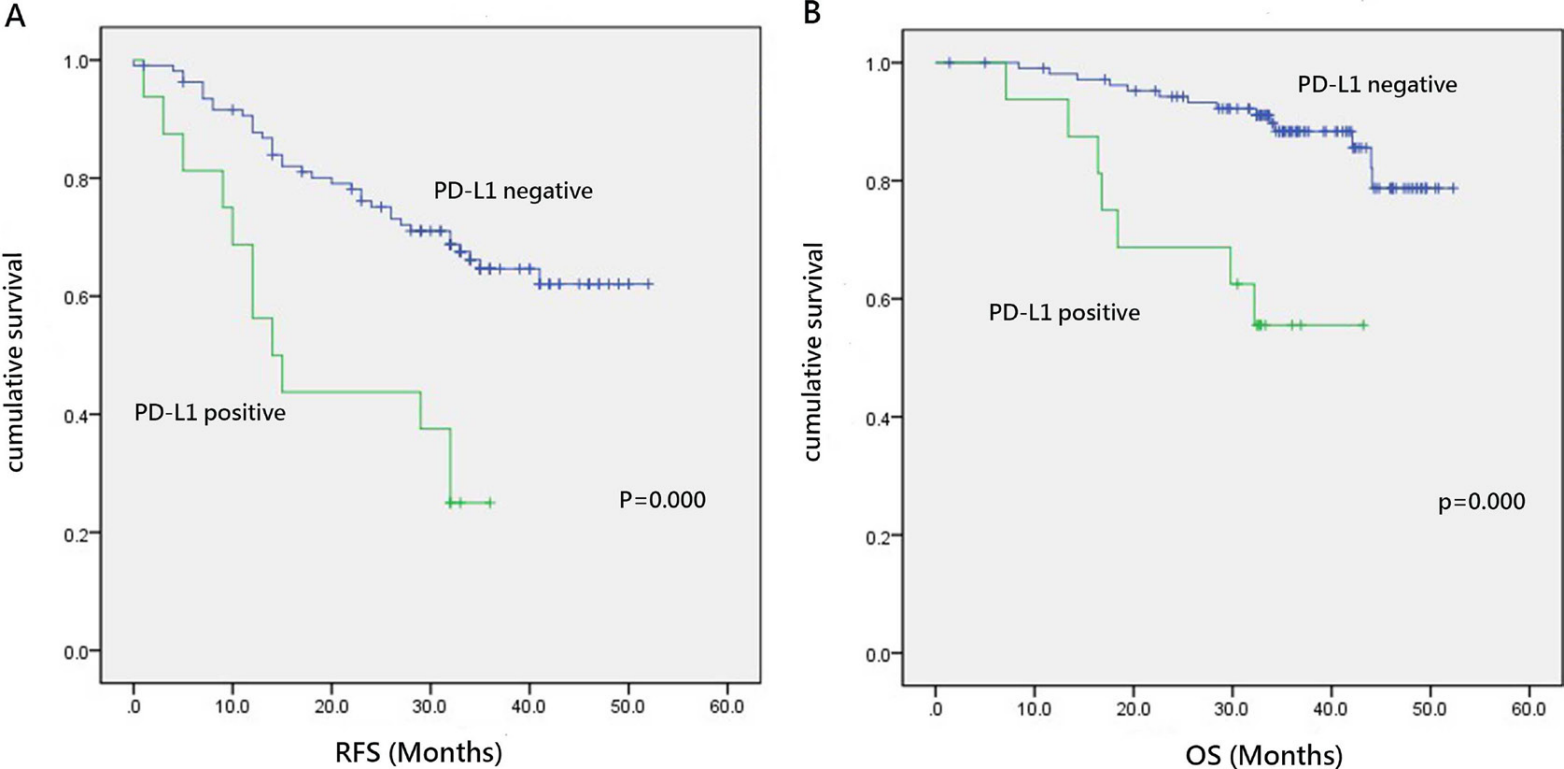
Prognostic significance of PD-L1 expression in lung adenocarcinoma (**A**) Recurrence free survival in patients with positive or negative programmed cell death ligand (PD-L1) expression. (**B**) Overall survival in patients with positive or negative PD-L expression.

In the 133 patients with lung adenocarcinoma, the median recurrence free survival (RFS) and overall survival (OS) times were 32.00 and 34.70 months, respectively. Forty-eight patients experienced recurrence at a median follow-up time of 14.00 months. Twenty-one patients died at a median follow up time of 22.60 months. Kaplan–Meier analysis revealed that PD-L1 expression was significantly associated with a shorter RFS (*P* = 0.000) and OS (*P* = 0.000) (Table 3, Figure 2). PD-L1 overexpression and advanced clinical stage were identified as independent prognostic factors in multivariate analyses (Table 4).

**Table 3:** Univariate analysis for recurrence free survival and overall survival

Variables	Median RFS (months)	*p*-value	Median OS (months)	*p*-value
**Age**		0.178		0.842
≥ 70	39.30		45.17	
< 70	36.04		46.31	
**Gender**		0.047		0.077
Female	40.19		42.82	
Male	32.25		47.99	
**Smoking**		0.138		0.292
Smokers	32.69		42.98	
Non- smokers	39.29		47.39	
**Tumor Size (cm)**		0.309		0.459
> 3	34.14		44.57	
≤ 3	39.08		47.36	
**Stage**		0.000		0.000
Early (I–II)	42.58		49.59	
Advanced (III–IV)	28.50		40.33	
**Invasion of pleural**		0.181		0.408
Yes	35.96		46.00	
No	40.25		46.54	
**Node metastasis**		0.032		0.066
Yes	32.18		42.55	
No	40.31		48.16	
**Post-operative therapy**		0.129		0.188
Yes	37.74		38.11	
No	35.81		36.41	
**PD-L1 expression**		0.000		0.000
Positive	19.88		32.61	
Negative	39.74		48.11	

Abbreviations: PD-L1, programmed cell death ligand 1; RFS, recurrence free survival; OS, overall survival.

**Table 4:** Multivariate analysis of prognostic factors of recurrence free survival and overall survival

Characteristic	Recurrence free survival		Overall survival	
	HR (95% CI)	*p*-value	HR (95% CI)	*p*-value
**PD-L1 expression**(Positive vs. negative)	2.522 (1.257–5.058)	0.009	3.395 (1.254–9.194)	0.016
**Gender**(Female vs male)	0.729 (0.403–1.321)	0.298	0.654 (0.262–1.632)	0.363
**Stage**(Advanced vs early)	2.939 (1.425–6.063)	0.004	3.688 (1.192–11.407)	0.023
**Node metastasis**(Yes vs. no)	0.881 (0.434–1.789)	0.725	0.976 (0.349–2.735)	0.964

Abbreviations: PD-L1, programmed cell death ligand 1.

## DISCUSSION

The clinical results show that monoclonal anti–PD-1/PD-L1 antibodies are powerful potential pharmaceutical therapy for advanced NSCLC. Careful selection of the targeted population is a prerequisite to protect patients from ineffective therapy and reduce side effects induced by the autoimmune system targeting the PD-1/PD-L1 axis. Preclinical data showed that PD-L1 expression is correlated with an increased likelihood of response to PD-L1 targeted therapy [10]. Establishing a reliable detection method to select the right patients is an urgent issue. Our study showed that the SP263 antibody is a good examination antibody, because of the high correlation of PD-L1 expression between the protein and mRNA level. PD-L1 is more highly expressed in male patients and smokers; in solid, papillary or micropapillary growth patterns compared to acinar and lepidic patterns; and its expression is an independent indicator of early disease recurrence and worse OS.

Evaluating human epidermal growth factor receptor-2 in breast cancer by IHC, which is economic and time-saving, has been successful. Efforts are being made to establish a method to evaluate PD-L1 expression by IHC. A multitude of detection PD-L1 IHC antibodies have been utilized, including the Ventana SP263 and SP142 clones, and the Dako 28–8 and 22C3 clones [11–13]. Various clones are currently being used in clinical trials to evaluate PD-L1 expression following use of different drugs. There is no consensus on the definition of a PD-L1 “positive” tumor, with different thresholds of cell expression used, e.g., 1%, 5%, 50%, and 25%. The lack of a standard examination method or a standard definition of a PD-L1 positive tumor following IHC staining is problematic. According to the central dogma of genetics, genes are first transcribed into mRNA and then translated into proteins. In the present study, the PD-L1 was detected and analyzed at the transcriptional level using mRNA ISH, and at the translational level using IHC. The concordance rate of the two methods was near perfect (95.5%; κ-coefficient, 0.824; *P* = 0.219). A recent study compared and validated 6 commercially available PD-L1 monoclonal antibodies (SP142, E1L3N, 9A11, SP263, 22C3, and 28–8). Their results showed that all 6 antibodies had high levels of concordance (*R*^2^ = 0.76–0.99). They further suggested that previously described differences in PD-L1 expression in tissue are independent of the antibody used and likely attributable to tumor heterogeneity, assay- or platform-specific variables, or other factors [14]. Nowadays, the National Comprehensive Cancer Network guidelines have been updated to include the treatment of lung cancer by anti PD-L1 therapy, clinicians should choose different drugs according to the approved accompanied diagnostic methods before definitive conclusions can be drawn.

The PD-L1 expression rate in lung adenocarcinoma samples in our study was about 13%. Previously reported expression rates of PD-L1 using different IHC antibodies range from 8% to 55% in lung adenocarcinoma or NSCLC patients [15–18]. This variation might be caused by the use of different antibodies, different interpretation criteria, and the lack of a consensus PD-L1 IHC method at present, or different population and tumor types. For example, in the Keynote-024 clinical trial which included 1653 patients, PD-L1 expression rate was 30.2% with a cut-off value of 50% or greater in NSCLC patients, which included adenocarcinoma and squamous cell carcinoma [19]. Our results are towards the lower limit of the previous results, mainly owing to ethnic group differences, and the relatively stricter evaluation criteria, i.e. only defining tumor positivity when more than 25% of the tumor cells were positive. In actuality, the clinical response rates in unselected patients across early trials have ranged from 10% to 20% in NSCLC [6, 8]. Further validation of this examination method and the clinical response is needed to test if this is suitable for selecting targeted patients.

In our study, we observed significant correlation of PD-L1 expression with clinicopathological characteristics. PD-L1 expression is associated with smoking habits. Smoking exposure may induce inflammatory as well as suppressive effects on the immune system [20]. Others have hypothesized that smoking-associated lung cancers have a higher mutational load, resulting in the creation of more tumor neoantigens and increased immunogenicity [21]. The results of the clinical trials showed that a history of smoking appears to impact the probability of response to anti–PD-1/PD-L1 monoclonal antibodies: the response rates in current/former smokers versus non/light smokers were associated with a stronger response to treatment [11, 13]. Besides smoking, our results also showed that PD-L1 expression is more frequent in male patients. The main reason for this is that male patients (31/53) were more likely to be smokers than the females (3/80). Other studies have also observed this correlation [22]. Previous studies have shown that overexpression of PD-L1 is much more common in poorly differentiated or higher pathological grades of lung adenocarcinoma [17, 23]. In our study, we also confirmed that papillary, micropapillary, and solid growth patterns are significantly associated with overexpression of PD-L1 compared to lepidic or acinar growth patterns. Besides these clinicopathological characteristics, others have also found that PD-L1 expression is associated with lymphovascular invasion, advanced lymph node stage, or pleural invasion, which we did not observe in our study [24].

Expression of PD-L1 was reported to be correlated with poor clinical outcomes in a number of human cancers, including lung, melanoma, breast, bladder, ovarian, pancreatic, esophageal adenocarcinoma, and kidney tumors [25]. In our study, we identified PD-L1 as an independent indicator of early recurrence and shorter survival duration in lung adenocarcinoma. Others have also observed an association of PD-L1 with poorer prognosis in lung adenocarcinoma. For example, Zhang et al. discovered that in lung adenocarcinoma both PD-L1 expression and PD-L2 expression were independent predictors of poor OS [26]. Chung et al. showed that PD-L1 and PD-L2 expression predicted poor prognosis for pulmonary adenocarcinoma [18]. The clinical trial results showed that PD-1 and PD-L1 blockade could prolong survival in patients with advanced lung cancer. All the above evidence indicated that overexpression of PD-L1 in tumor cells can help the tumor cells to escape the surveillance of T cells, in turn acquiring resistance and becoming more prone to proliferation or metastasis.

In conclusion, we have demonstrated that expression of PD-L1 was present in 13.5% of lung adenocarcinoma patients and was associated with a poorer prognosis. Further studies are warranted to confirm PD-L1 expression, and the therapeutic effect of PD-1/PD-L1 blockade, in clinical trials.

## MATERIALS AND METHODS

### Patient information

One hundred and thirty-three patients were enrolled in this study, and underwent a surgical operation with a final diagnosis of lung adenocarcinoma at Peking Union Medical College Hospital (PUMCH, Beijing, China) between January 2012 and December 2015. For poorly differentiated cases, additional immunostaining for Napsin A and thyroid transcription factor 1 (TTF-1) was performed to confirm the final results.

Clinical information for the patients was retrieved from the PUMCH digital database, including age, gender, smoking habit, clinical stage, treatment methods, RFS, and OS. Clinical stage was determined at the time of surgery, according to the American Joint Commission on Cancer (AJCC), 7th edition, tumor-node-metastasis staging system. RFS was defined as the time from operation to relapse or until the endpoint of the study. OS was calculated as the time from surgery to death or until the endpoint of the research, which was April 30^th^, 2016.

For each tumor sample, hematoxylin-eosin staining (HE) slides were all re-reviewed by two experienced pathologists to confirm the final diagnosis. The growth pattern of lung adenocarcinoma was defined according to the guidelines of the 2011 International Association for the Study of Lung Cancer/American Thoracic Society/European Respiratory Society classification system that includes lepidic, acinar, papillary, micropapillary, and solid predominant patterns. Both pathologists had no knowledge of the clinical information or PD-L1 expression status of these cases during the review. Additionally, the following pathological characteristics were evaluated: tumor size, pleural invasion, and node metastasis status.

This study was approved by the Institutional Review Board of Peking Union Medical College Hospital. Informed consent was obtained from all patients before they underwent the operation.

### Tissue microarray construction

The selected area of representative morphology was labeled on the HE slides. The corresponding formalin-fixed paraffin-embedded (FFPE) primary tumor specimens were obtained from the Department of Pathology. A tissue microarray (TMA) construction machine (Tissue Array minicore, Mitogen, England) was used. Three core-tissue biopsies, 1.0 mm in diameter, were collected for each case.

### PD-L1 immunohistochemical examination

The IHC study was performed on formalin-fixed, paraffin-embedded TMA sections, with a thickness of 4 μm, using standard autostaining protocols on a Ventana Benchmark ULTRA autostainer (Ventana Medical Systems, Inc., Tucson, AZ, USA). The PD-L1 antibody used in our study was SP263 (Roche Ventana, Tucson, AZ, USA). Tumors with positive membrane staining in more than 25% of the whole tumor were deemed positive, as suggested in a study by Ratcliffe et al. [27].

### PD-L1 mRNA detection by *in situ* hybridization

For detection of PD-L1 expression, at the mRNA level, we used *in situ* hybridization (ISH). An RNAscope FFPE 2.0 HD detection kit (Brown, Advanced Cell Diagnostics, Hayward, CA, USA) was used according to the manufacturer’s instructions. Briefly, 5 μm-sections were deparaffinized, boiled with pre-amplification reagent for 15 minutes and submitted to protease digestion followed by hybridization for 2 h with a mixture containing target probes against human PD-L1, ubiquitin C (UBC) as a positive control, and the bacterial gene DapB as a negative control. Hybridization signals were detected with 3,3′-diaminobenzidine. Positive staining was indicated by brown punctate dots in the cytoplasm or nucleus. PD-L1 mRNA expression levels were categorized into 5 grades according to the manufacture’s scoring guideline: score 0, no staining or < 1 dot per cell; score 1, 1–3 dots per cell (visible at 20–40X); score 2, 4–10 dots per cell (visible at 20–40X); score 3, > 10 dots per cell and < 10% positive cells have dot clusters (visible at 20–40X); score 4, > 10 dots per cell and > 10% positive cells have dot clusters (visible at 20–40X). We grouped score 0, 1, 2 as negative, 3 and 4 as positive.

### Statistical analysis

The relationship between PD-L1 expression and clinicopathological characteristics was analyzed using the chi-squared test. Survival analysis was calculated by the Kaplan-Meier method, the log rank test was used to compare the difference of two groups, and multivariate analysis using the Cox regression model was conducted to analyze the related clinicopathological features. All tests were 2-sided. P < 0.05 was considered statistically significant. The statistical analyses were performed using SPSS software (version 21; SPSS, Chicago, IL, USA).

For analysis of concordance between the results of IHC and RNA ISH, the kappa value was calculated. The significance of the *κ*-value was considered as follows: value ≤ 0.40, poor to fair concordance; 0.41–0.60, moderate concordance; 0.61–0.80, substantial concordance; 0.81–1.00, almost perfect concordance.

## Abbreviations

NSCLC: non-small cell lung cancer
EGFR: epidermal growth factor receptor
ALK: anaplastic lymphoma kinase
TILs: tumor-infiltrating lymphocytes
Programmed cell death protein 1: PD-1: Programmed cell death protein 1
PD-L1: Programmed cell death ligand 1
IHC: Immunohistochemistry
RFS: Recurrence free survival
OS: Overall survival
FFPE: Formalin-fixed paraffin-embedded
TMA: Tissue microarray.

## References

[1] Saito M, Shiraishi K, Kunitoh H, Takenoshita S, Yokota J, Kohno T (2016). Gene aberrations for precision medicine against lung adenocarcinoma. Cancer Sci.

[2] Devarakonda S, Morgensztern D, Govindan R (2015). Genomic alterations in lung adenocarcinoma. Lancet Oncol.

[3] Yu HA, Tian SK, Drilon AE, Borsu L, Riely GJ, Arcila ME, Ladanyi M (2015). Acquired Resistance of EGFR-Mutant Lung Cancer to a T790M-Specific EGFR Inhibitor: Emergence of a Third Mutation (C797S) in the EGFR Tyrosine Kinase Domain. JAMA Oncol.

[4] Xu Y, Liu H, Chen J, Zhou Q (2010). Acquired resistance of lung adenocarcinoma to EGFR-tyrosine kinase inhibitors gefitinib and erlotinib. Cancer Biol Ther.

[5] Kumarakulasinghe NB, van Zanwijk N, Soo RA (2015). Molecular targeted therapy in the treatment of advanced stage non-small cell lung cancer (NSCLC). Respirology.

[6] Brahmer JR, Tykodi SS, Chow LQ, Hwu WJ, Topalian SL, Hwu P, Drake CG, Camacho LH, Kauh J, Odunsi K, Pitot HC, Hamid O, Bhatia S (2012). Safety and activity of anti-PD-L1 antibody in patients with advanced cancer. N Engl J Med.

[7] Califano R, Kerr K, Morgan RD, Russo GL, Garassino M, Morgillo F, Rossi A (2016). Immune Checkpoint Blockade: A New Era for Non-Small Cell Lung Cancer. Curr Oncol Rep.

[8] Topalian SL, Hodi FS, Brahmer JR, Gettinger SN, Smith DC, McDermott DF, Powderly JD, Carvajal RD, Sosman JA, Atkins MB, Leming PD, Spigel DR, Antonia SJ (2012). Safety, activity, and immune correlates of anti-PD-1 antibody in cancer. N Engl J Med.

[9] Sznol M, Chen L (2013). Antagonist antibodies to PD-1 and B7-H1 (PD-L1) in the treatment of advanced human cancer—response. Clin Cancer Res.

[10] Pardoll DM (2012). The blockade of immune checkpoints in cancer immunotherapy. Nat Rev Cancer.

[11] Gettinger SN, Horn L, Gandhi L, Spigel DR, Antonia SJ, Rizvi NA, Powderly JD, Heist RS, Carvajal RD, Jackman DM, Sequist LV, Smith DC, Leming P (2015). Overall Survival and Long-Term Safety of Nivolumab (Anti-Programmed Death 1 Antibody, BMS-936558, ONO-4538) in Patients With Previously Treated Advanced Non-Small-Cell Lung Cancer. J Clin Oncol.

[12] Garon EB, Rizvi NA, Hui R, Leighl N, Balmanoukian AS, Eder JP, Patnaik A, Aggarwal C, Gubens M, Horn L, Carcereny E, Ahn MJ, Felip E (2015). Pembrolizumab for the treatment of non-small-cell lung cancer. N Engl J Med.

[13] Herbst RS, Soria JC, Kowanetz M, Fine GD, Hamid O, Gordon MS, Sosman JA, McDermott DF, Powderly JD, Gettinger SN, Kohrt HE, Horn L, Lawrence DP (2014). Predictive correlates of response to the anti-PD-L1 antibody MPDL3280A in cancer patients. Nature.

[14] Gaule P, Smithy JW, Toki M, Rehman J, Patell-Socha F, Cougot D, Collin P, Morrill P, Neumeister V, Rimm DL (2016). A Quantitative Comparison of Antibodies to Programmed Cell Death 1 Ligand 1. JAMA Oncol.

[15] Azuma K, Ota K, Kawahara A, Hattori S, Iwama E, Harada T, Matsumoto K, Takayama K, Takamori S, Kage M, Hoshino T, Nakanishi Y, Okamoto I (2014). Association of PD-L1 overexpression with activating EGFR mutations in surgically resected nonsmall-cell lung cancer. Ann Oncol.

[16] D’Incecco A, Andreozzi M, Ludovini V, Rossi E, Capodanno A, Landi L, Tibaldi C, Minuti G, Salvini J, Coppi E, Chella A, Fontanini G, Filice ME (2015). PD-1 and PD-L1 expression in molecularly selected non-small-cell lung cancer patients. Br J Cancer.

[17] Yang CY, Lin MW, Chang YL, Wu CT, Yang PC (2014). Programmed cell death-ligand 1 expression in surgically resected stage I pulmonary adenocarcinoma and its correlation with driver mutations and clinical outcomes. Eur J Cancer.

[18] Chung DH, Kerr KM, Tsao MS, Nicholson AG, Yatabe Y, Wistuba II, Hirsch FR (2015). Programmed Death-Ligand 1 Immunohistochemistry in Lung Cancer: In what state is this art?. Mod Pathol.

[19] Reck M, Rodriguez-Abreu D, Robinson AG, Hui R, Csoszi T, Fulop A, Gottfried M, Peled N, Tafreshi A, Cuffe S, O’Brien M, Rao S, Hotta K (2016). Pembrolizumab versus Chemotherapy for PD-L1-Positive Non-Small-Cell Lung Cancer. N Engl J Med.

[20] Vassallo R, Tamada K, Lau JS, Kroening PR, Chen L (2005). Cigarette smoke extract suppresses human dendritic cell function leading to preferential induction of Th-2 priming. J Immunol.

[21] Lawrence MS, Stojanov P, Polak P, Kryukov GV, Cibulskis K, Sivachenko A, Carter SL, Stewart C, Mermel CH, Roberts SA, Kiezun A, Hammerman PS, McKenna A (2013). Mutational heterogeneity in cancer and the search for new cancer-associated genes. Nature.

[22] Cha YJ, Kim HR, Lee CY, Cho BC, Shim HS (2016). Clinicopathological and prognostic significance of programmed cell death ligand-1 expression in lung adenocarcinoma and its relationship with p53 status. Lung Cancer.

[23] Koh J, Go H, Keam B, Kim MY, Nam SJ, Kim TM, Lee SH, Min HS, Kim YT, Kim DW, Jeon YK, Chung DH (2015). Clinicopathologic analysis of programmed cell death-1 and programmed cell death-ligand 1 and 2 expressions in pulmonary adenocarcinoma: comparison with histology and driver oncogenic alteration status. Mod Pathol.

[24] Takada K, Okamoto T, Shoji F, Shimokawa M, Akamine T, Takamori S, Katsura M, Suzuki Y, Fujishita T, Toyokawa G, Morodomi Y, Okano S, Oda Y (2016). Clinical Significance of PD-L1 Protein Expression in Surgically Resected Primary Lung Adenocarcinoma. J Thorac Oncol.

[25] Zou W, Chen L (2008). Inhibitory B7-family molecules in the tumour microenvironment. Nat Rev Immunol.

[26] Zhang Y, Wang L, Li Y, Pan Y, Wang R, Hu H, Li H, Luo X, Ye T, Sun Y, Chen H (2014). Protein expression of programmed death 1 ligand 1 and ligand 2 independently predict poor prognosis in surgically resected lung adenocarcinoma. Onco Targets Ther.

[27] Ratcliffe Marianne J, Sharpe Alan, Midha Anita, Barker Craig, Scott Marietta, Scorer Paul, Walker J (2016). A Comparative Study of PD-L1 Diagnostic Assays and the Classification of Patients as PD-L1 Positive and PD-L1 Negative. American Association for Cancer Research (AACR) Annual Meeting.

